# Recessive *PKD1* Mutations Are Associated With Febrile Seizures and Epilepsy With Antecedent Febrile Seizures and the Genotype-Phenotype Correlation

**DOI:** 10.3389/fnmol.2022.861159

**Published:** 2022-05-10

**Authors:** Jing-Yang Wang, Jie Wang, Xin-Guo Lu, Wang Song, Sheng Luo, Dong-Fang Zou, Li-Dong Hua, Qian Peng, Yang Tian, Liang-Di Gao, Wei-Ping Liao, Na He

**Affiliations:** ^1^Department of Neurology, Institute of Neuroscience, The Second Affiliated Hospital of Guangzhou Medical University, Guangzhou, China; ^2^Key Laboratory of Neurogenetics and Channelopathies of the Ministry of Education of China, Guangzhou, China; ^3^Epilepsy Center, Department of Neurology, Shenzhen Children’s Hospital, Shenzhen, China; ^4^Translational Medicine Center, Guangdong Women and Children Hospital, Guangzhou, China; ^5^Department of Pediatrics, Dongguan City Maternal and Child Health Hospital, Southern Medical University, Dongguan, China; ^6^Department of Neurology, Guangzhou Women and Children’s Medical Center, Guangzhou Medical University, Guangzhou, China

**Keywords:** *PKD1* gene, epilepsy with antecedent febrile seizures, genotype-phenotype association, monoallelic mutation, compound heterozygous mutations

## Abstract

**Objective:**

The *PKD1* encodes polycystin-1, a large transmembrane protein that plays important roles in cell proliferation, apoptosis, and cation transport. Previous studies have identified *PKD1* mutations in autosomal dominant polycystic kidney disease (ADPKD). However, the expression of *PKD1* in the brain is much higher than that in the kidney. This study aimed to explore the association between *PKD1* and epilepsy.

**Methods:**

Trios-based whole-exome sequencing was performed in a cohort of 314 patients with febrile seizures or epilepsy with antecedent febrile seizures. The damaging effects of variants was predicted by protein modeling and multiple *in silico* tools. The genotype-phenotype association of *PKD1* mutations was systematically reviewed and analyzed.

**Results:**

Eight pairs of compound heterozygous missense variants in *PKD1* were identified in eight unrelated patients. All patients suffered from febrile seizures or epilepsy with antecedent febrile seizures with favorable prognosis. All of the 16 heterozygous variants presented no or low allele frequencies in the gnomAD database, and presented statistically higher frequency in the case-cohort than that in controls. These missense variants were predicted to be damaging and/or affect hydrogen bonding or free energy stability of amino acids. Five patients showed generalized tonic-clonic seizures (GTCS), who all had one of the paired missense mutations located in the PKD repeat domain, suggesting that mutations in the PKD domains were possibly associated with GTCS. Further analysis demonstrated that monoallelic mutations with haploinsufficiency of *PKD1* potentially caused kidney disease, compound heterozygotes with superimposed effects of two missense mutations were associated with epilepsy, whereas the homozygotes with complete loss of *PKD1* would be embryonically lethal.

**Conclusion:**

*PKD1* gene was potentially a novel causative gene of epilepsy. The genotype-phenotype relationship of *PKD1* mutations suggested a quantitative correlation between genetic impairment and phenotypic variation, which will facilitate the genetic diagnosis and management in patients with *PKD1* mutations.

## Introduction

*PKD1* (MIM 601313) is a gene with 46 exons located on chromosome 16p13. The encoded protein polycystin-1 (PC1) forms a complex with polycystin-2 (PC2), which regulates calcium permeable cation channels and intracellular calcium homeostasis (Semmo et al., 2014; Kim et al., 2016; Su et al., 2018). PC1 is distributed widely in multiple tissues, especially highly in the brain (Fagerberg et al., 2014). Its expression is also developmentally regulated with the highest level in fetal life and maintained throughout adulthood (Geng et al., 1997). Homozygous *Pkd1* knockout mice have exhibited neural tube defects and embryonic or perinatal lethality (Lu et al., 2001; Lantinga-van Leeuwen et al., 2007). PC1 can also interact with homer 1/Vesl-1 and plays a role in synaptic plasticity in postnatal mouse hippocampus (Stokely et al., 2006). So far, *PKD1* mutations were reported to be associated with autosomal dominant polycystic kidney disease (ADPKD, MIM 173900), which was featured by dilatation of the renal tubules leading to cyst formation and progressive renal failure. The relationship between *PKD1* and diseases of the brain remains unknown.

Febrile seizures (FS) are the most common human convulsive event. Children with FS have a higher risk of developing spontaneous afebrile seizures (Baulac et al., 2004). Retrospective studies indicated that 10–15% of patients with epilepsy had antecedent FS (epilepsy with febrile seizures plus, EFS+) (Baulac et al., 2004). The gene associated with FS/EFS+ include *ADGRV1, CPA6, DYRK1A, FEB2, FEB5, FEB6, FEB7, FEB9, FEB10, FGF13, GABRB3, GABRD, GABRG2, GEFSP4, GEFSP6, GEFSP8, HCN1, NPRL3, SCN1A, SCN1B, SCN2A, SCN9A, and STX1B* (OMIM)^1^. In this study, we performed trios-based whole-exome sequencing (WES) in a cohort of patients of FS and epilepsy with antecedent FS. *PKD1* compound heterozygous mutations were identified in eight unrelated cases, among which seven probands presented epilepsy with antecedent FS. This study suggested that *PKD1* gene is potentially a candidate pathogenic gene of FS and epilepsy with antecedent FS.

## Materials and Methods

### Patients

A total of 314 cases with febrile seizures and epilepsy with antecedent FS were recruited for genetic screening during January 2018–July 2021 from four hospitals, including The Second Affiliated Hospital of Guangzhou Medical University, Shenzhen Children’s Hospital, Guangzhou Women and Children’s Medical Center of Guangzhou Medical University, and Dongguan City Maternal and Child Health Hospital. Clinical characteristics of the affected individuals were collected, including present age, gender, age at seizure onset, seizure course, family history, state of development, effective antiepileptic drugs (AEDs). Magnetic resonance imaging (MRI) scans were performed to detect any brain structure abnormalities. Long-term video-EEG examination was obtained that included open-close eyes test, intermittent photic stimulation, hyperventilation, and sleeping recording. The results were reviewed by two qualified electroencephalographers. Epileptic seizures and epilepsies were diagnosed according to the criteria of the Commission on Classification and Terminology of the ILAE (1981, 1989, 2001, 2010, and 2017). FS was diagnosed with the criteria: (1) a seizure occurring in childhood after age of 1 month to 6 years accompanied by a fever, (2) the febrile illness not caused by central nervous system infection, (3) not meeting criteria for other acute symptomatic seizures. The patients and their parents (trios), and other available family members were screened for genetic variants by the whole-exome sequencing.

For the controls, a cohort of 296 healthy Chinese volunteers was recruited as a normal control group as our previous report (Wang et al., 2021). Frequencies of the identified variants were also compared with that in the other control populations, including East Asian and general populations in the Genome Aggregation Database (gnomAD)^2^.

This study was approved by the Ethics Committee of The Second Affiliated Hospital of Guangzhou Medical University, and written informed consent was obtained from all patients and their parents.

### Whole Exome Sequencing

Whole blood samples were collected from the probands, their parents, and other available family members. Qiagen Flexi Gene DNA kit (Qiagen, Hilden, Germany) was used to extract genomic DNA from the whole blood samples. Trio-based whole-exome sequencing was performed on an Illumina HiSeq 2000 system by BGI-Shenzhen (Shenzhen, China) as previously described (Wang et al., 2021). The sequencing data were generated by massively parallel sequencing with >125 times average depth and >98% coverage in the capture region of the chip to obtain high-quality reads that were mapped to the Genome Reference Consortium Human Genome build 37 (GRCh37) by Burrows-Wheeler alignment. Single-nucleotide point variants and indels were called with the Genome Analysis Toolkit. To obtain the comprehensive list of candidate pathogenic variants in each trio, we adopted a case-by-case analytical pattern. We first prioritized the rare variants with a minor allele frequency <0.005 in the 1000 Genomes Projects, Exome Aggregation Consortium, and gnomAD, and then screened for possibly disease-causing variants in each case under the following five models: (1) epilepsy-associated gene (Wang et al., 2017); (2) *de novo* variant dominant; (3) autosomal recessive inheritance, including homozygous and compound heterozygous variants; (4) X-linked; and (5) co-segregation analysis. To identify novel epilepsy-associated genes, we put the known epilepsy-associated genes aside (Wang et al., 2017). Genes with repetitively identified *de novo* variants, bi-allelic variants, hemizygous variants, or variants with segregations, were selected for further studies to define the gene-disease association. *PKD1* appeared as a candidate gene with recurrent compound heterozygous variants in this cohort. Sanger sequencing was used to validate the positive findings and the variant origination. All *PKD1* variants identified in this study were annotated to reference transcript NM_001009944.2.

### Protein Modeling and Mutation Analysis

To evaluate the pathogenicity of candidate variants, the structure of PC1 was modeled to predict the effect of missense mutations on protein structure by using the Iterative Threading ASSEmbly Refinement software (I-TASSER)^3^. The confidence of models was quantitatively measured by a C-score in the range of [–5,2]. Three models were predicted and utilized in this study with C-scores of −0.64, −1.14, and −3.02, respectively. PyMOL 2.3 software was used for three-dimensional protein structure visualization and analysis.

To evaluate the protein stability changes upon single nucleotide mutations, the free energy change value (ΔΔG, kcal/mol) was predicted by I-Mutant server^4^ (Capriotti et al., 2005). Variants were divided into three classes: large increase of protein stability (ΔΔG > 0.5 kcal/mol), large decrease of protein stability (ΔΔG < −0.5 kcal/mol), and neutral stability (−0.5 kcal/mol ≤ ΔΔG ≤ 0.5 kcal/mol). The consequences of all the missense variants were predicted by 21 *in silico* tools, including SIFT, PolyPhen2_HVAR, CADD, MutationTaster, Fathmm-MKL, fitCons, PhastCons, and GERP++.

### Genotype-Phenotype Association Analysis

Previously, *PKD1* mutations were reported to be associated with ADPKD. To explore the genotype-phenotype association of *PKD1* mutations, we systematically reviewed all *PKD1* mutations in the HGMD database (version: HGMD Professional 2021.3)^5^ and the ADPKD Variant database (version 4.0)^6^. The registered variants were reviewed from the references indexed in the databases. The literature was also searched on the PubMed database using the following search items: “biallelic mutation *PKD1*,” “compound heterozygous mutation *PKD1*,” and “homozygous mutation *PKD1.*” Some biallelic *PKD1* mutations were reviewed from the cited references of publications from the initial search using the above-mentioned terms. Monoallelic and biallelic *PKD1* mutations identified in patients with ADPKD or sporadic PKD were systematically reviewed and classified.

### Statistical Analysis

R statistical software (version 4.0.3) was used for data processing. The frequencies of the *PKD1* variants between the epilepsy cohort and the controls were compared by a two-sided Fisher exact test (Consortium, 2015). The burden of recessive variants was analyzed according to the method recommended recently (Martin et al., 2018). A *p* value of <0.05 was considered to be statistically significant.

### Evaluating *PKD1* Gene as a Novel Candidate Epilepsy Gene

The Clinical Validity Framework that was developed by Clinical Genome Resource (ClinGen) was performed to evaluate *PKD1* as a novel candidate epilepsy gene (Strande et al., 2017).

## Results

### Identification of *PKD1* Mutations

**FIGURE 1:**
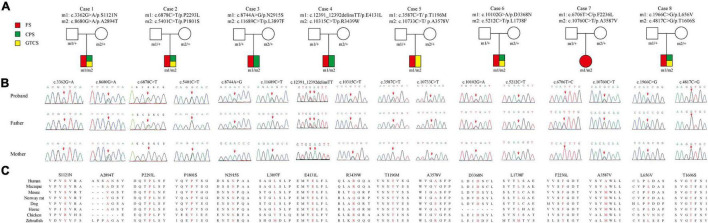
Genetic data of cases with *PKD1* mutations. **(A)** Pedigrees of eight cases with compound heterozygous *PKD1* missense mutations and their corresponding phenotypes. FS, febrile seizure; CPS, complex partial seizure; GTCS, generalized tonic-clonic seizure. **(B)** DNA sequencing chromatogram of *PKD1* mutations. Arrows indicate the positions of the mutations. **(C)** Amino acid sequence alignment of the sixteen missense variants show that residues P2293L, N2915S, E4131L, D3368N, and T1606S are highly conserved in various species; S1121N, P1801S, L3897F, T1196M, L1738F, and F2236L were likely to be highly conserved across mammalian species; while L656V, A2894T, R3439W, A3578V, and A3587V are less conserved.

Eight pairs of compound heterozygous missense variants, including c.3362G > A/p.S1121N and c.8680G > A/p.A2894T, c.5401C > T/p.P1801S and c.6878C > T/p.P2293L c.8744A > G/p.N2915S and c.11689C > T/p.L3897F, c.10315C > T/p.R3439W and c.12391_12392delinsTT/p.E4131L, c.3587C > T/p.T1196M and c.10733C > T/p.A3578V, c.5212C > T/p.L1738F and c.10102G > A/p.D3368N, c.6706T > C/p.F2236L and c.10760C > T/p.A3587V, and c.1966C > G/p.L656V and c.4817C > G/p.T1606S, were identified in eight unrelated cases with FS or EFS+ (Table 1 and Figure 1). The heterozygotes were inherited from their asymptomatic parents, indicating that *PKD1* variants in FS and EFS+ followed an autosomal recessive mode of inheritance.

**TABLE 1:** Clinical feature of the individuals with *PKD1* mutations.

Case	Variants	Sex	Age (year)	Onset (year)	Seizure course	EEG	Brain MRI	Development	Treatment (AEDs)	Seizure-free duration	Diagnosis
Case 1	c.3362G > A/p.S1121N c.8680G > A/p.A2894T	Male	10	6	FS, CPS, GTCS (twice a year)	Diffused spikes and spike-slow waves, obviously in the foreheads	Normal	Normal	LEV	1 year	EFS+
Case 2	c.5401C > T/p.P1801S c.6878C > T/p.P2293L	Male	3.5	2.5	FS, CPS, GTCS (twice a month)	Spikes in the bilateral frontal and central regions	Normal	Normal	–	6 months	EFS+
Case 3	c.8744A > G/p.N2915S c.11689C > T/p.L3897F	Male	10	4	FS, CPS (three times a year)	Spike-slow and slow waves in right frontal and temporal regions	Normal	Normal	OXC, LTG	1 year	EFS+
Case 4	c.10315C > T/p.R3439W c.12391_12392delinsTT/p.E4131L	Male	11	1	FS, CPS (5–6 times a month)	Bilateral occipital spike-slow and slow waves; diffused spike-slow waves	Normal	Normal	CNZ, LEV	1 year	EFS+
Case 5	c.3587C > T/p.T1196M c.10733C > T/p.A3578V	Male	4.5	3	FS, GTCS (four times a month)	Irregular diffused spike-slow waves, obviously in the right areas	Normal	Normal	VPA	5 months	EFS+
Case 6	c.5212C > T/p.L1738F c.10102G > A/p.D3368N	Male	2.5	1	FS, CPS, GTCS (four times a month)	Spikes and spike-slow waves in the frontal, central, and temporal regions	Normal	Normal	VPA	3 months	EFS+
Case 7	c.6706T > C/p.F2236L c.10760C > T/p.A3587V	Female	9	2	FS (twice)	NA	NA	Normal	–	2 years	FS
Case 8	c.1966C > G/p.L656V c.4817C > G/p.T1606S	Male	6	3	FS, CPS, GTCS (once a month)	Spike-slow waves in the right frontal and temporal regions	Normal	Normal	LTG	3 months	EFS+

*AEDs, antiepileptic drugs; FS, febrile seizure; CNZ, clonazepam; CPS, complex partial seizure; EEG, electroencephalogram; EFS+, epilepsy with antecedent FS; GTCS, generalized tonic-clonic seizure; LEV, levetiracetam; LTG, lamotrigine; MRI, magnetic resonance imaging; NA, not available; OXC, oxcarbazepine; VPA, valproate.*

All heterozygous variants presented no or low allele frequencies in the gnomAD database (Table 2). Homozygotes of these variants were not listed in any public databases. None of the variants were observed in the 296 normal controls. When the burden of recessive variants was analyzed (Martin et al., 2018), the *PKD1* variants in the present cohort were significantly more than the expected number by chance in the East Asian population [minor allele frequency (MAF) < 0.005, *p* = 0.0004208]. Furthermore, the aggregate frequency of the variants in this cohort was significantly higher than that in the five controls (Table 2), including the gnomAD-all population (16/628 vs. 248/221464, *p* = 2.2 × 10^–16^), the controls of gnomAD-all population (vs. 112/88270, *p* = 1.43 × 10^–15^), the gnomAD-East Asian population (vs. 162/16258, *p* = 0.001014), the controls of the gnomAD-East Asian population (vs. 76/7610, *p* = 0.002089), and the 296 normal controls (vs. 0/296, *p* = 3.06 × 10^–5^). None of the eight affected patients had pathogenic or likely pathogenic mutations in the 977 genes known to be associated with epileptic phenotypes (Wang et al., 2017).

**TABLE 2:** A gene-based burden analysis for *PKD1* mutations identified in this study.

	Allele count/number in this study	Allele count/number in the gnomAD-all population	Allele count/number in the controls of gnomAD-all population	Allele count/number in the gnomAD-East Asian	Allele count/number in the controls of gnomAD-East Asian	Allele count/number in the controls of 296 healthy volunteers
**Identified *PKD1* mutations**						
chr16: 2161806 (c.3362G > A/p.S1121N)	1/628	–/–	–/–	–/–	–/–	0
chr16: 2153378 (c.8680G > A/p.A2894T)	1/628	17/267314 (0.00006360)	9/101036 (0.00008908)	1/19432 (0.00005146)	0/8668 (0)	0
chr16: 2159767 (c.5401C > T/p.P1801S)	1/628	8/239710 (0.00003337)	4/106082 (0.00003771)	4/17982 (0.0002224)	3/8750 (0.0003429)	0
chr16: 2158290 (c.6878C > T/p.P2293L)	1/628	33/275052 (0.0001200)	18/118012 (0.0001525)	19/19500 (0.0009744)	10/9576 (0.001044)	0
chr16: 2153314 (c.8744A > G/p.N2915S)	1/628	39/272678 (0.0001430)	18/116880 (0.0001540)	21/19496 (0.001077)	10/9604 (0.001041)	0
chr16: 2141447 (c.11689C > T/p.L3897F)*	1/628	4/4272 (0.0009363)	2/2036 (0.0009823)	0/62 (0)	0/40 (0)	0
chr16: 2147410 (c.10315C > T/p.R3439W)	1/628	51/275532 (0.0001851)	20/116618 (0.0001715)	39/19728 (0.001977)	18/9778 (0.001841)	0
chr16: 2140338, 2140339 (c.12391_12392delinsTT/p.E4131L)	1/628	–/–	–/–	–/–	–/–	0
chr16: 2161581 (c.3587C > T/p.T1196M)*	1/628	4/181588 (0.00002203)	3/78930 (0.00003801)	3/13498 (0.0002223)	3/5936 (0.0005054)	0
chr16: 2143900 (c.10733C > T/p.A3578V)*	1/628	5/226808 (0.00002205)	2/80606 (0.00002481)	2/16796 (0.0001191)	1/7018 (0.0001425)	0
chr16: 2159956 (c.5212C > T/p.L1738F)	1/628	5/239702 (0.00002086)	5/105408 (0.00004743)	5/20568 (0.0002431)	5/13110 (0.0003814)	0
chr16: 2147934 (c.10102G > A/p.D3368N)	1/628	63/251880 (0.0002501)	24/108086 (0.0002220)	57/18150 (0.003140)	22/8750 (0.002514)	0
chr16: 2158462 (c.6706T > C/p.F2236L)	1/628	5/246952 (0.00002025)	2/108614 (0.00001841)	5/17974 (0.0002782)	2/8674 (0.0002306)	0
chr16: 2143873 (c.10760C > T/p.A3587V)	1/628	8/221464 (0.00003612)	3/88270 (0.00003399)	0/16258 (0)	0/7610 (0)	0
chr16: 2165510 (c.1966C > G/p.L656V)	1/628	–/–	–/–	–/–	–/–	0
chr16: 2160351 (c.4817C > G/p.T1606S)	1/628	6/249556 (0.00002404)	2/109386 (0.00001828)	6/18364 (0.0003267)	2/9044 (0.0002211)	0
**Total**	16/628	248/221464 (0.001120)	112/88270 (0.001269)	162/16258 (0.009964)	76/7610 (0.009987)	0
***P* value**		2.2 × 10^–16^	1.43 × 10^–15^	0.001014	0.002089	3.06 × 10^–5^
**OR (95% CI)**		23.327 (13.042–38.887)	20.578 (11.300–35.177)	2.597 (1.441–4.382)	2.591 (1.401–4.518)	∞ (3.705–∞)

*OR, odd ratio; P values and odd ratio were estimated by two-sided Fisher’s exact test.*

*The variants were excluded from statistical analysis because of the low-quality genotypes in gnomAD.

The missense mutations P2293L, N2915S, E4131L, D3368N, and T1606S affected amino acid residues that are highly conserved in various species, and the residues S1121N, P1801S, L3897F, T1196M, L1738F, and F2236L were likely to be highly conserved across mammalian species, while the missense mutation L656V, A3578V, and A3587V were less conserved by sequence alignment, but were predicted to be conserved by *in silico* tools (Supplementary Table 1).

### Molecular Location and Effect of *PKD1* Variants

**FIGURE 2:**
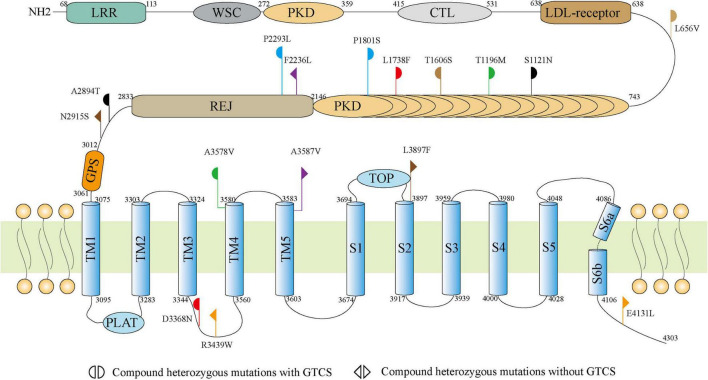
Schematic illustration of the PC1 and locations of the *PKD1* mutations identified in this study.

The 4303-residue PC1 comprises a long N-terminal extracellular region, eleven transmembrane helices, and a C-terminal coiled-coil domain^7^. The N-terminal extracellular portion contains leucine-rich repeats (LRR), a C-type lectin domain, immunoglobulin (Ig)-like PKD repeat domains, a single low-density lipoprotein (LDL) receptor motif, the receptor for egg jelly (REJ) domain, and G protein-coupled receptor proteolysis site (GPS; Hughes et al., 1995; Rondeau, 1995; Gallagher et al., 2010; Arac et al., 2012; Ha et al., 2020). The majority (10/16) of missense mutations were located in the N-terminal extracellular region, among which five variants were located in the PKD repeat domains, including S1121N, T1196M, T1606S, L1738F, and P1801S. This region of PC1 likely plays an important role in cell-cell and cell-matrix interactions (Babich et al., 2004). The other six variants were located in transmembrane helices and C-terminal coiled-coil domain, including R3439W and L3368F in TM3–TM4 linker, A3578V in TM4 domain, A3587V in TM5 domain, L3897F in S1–S2 linker and E4131L in C-terminal (Figure 2). No hotspot variant or region was observed.

**FIGURE 3:**
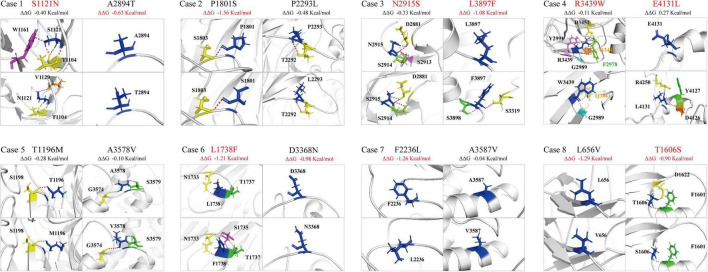
Hydrogen bond changes of the *PKD1* mutants. Variants with changes on hydrogen bonding or free energy stability are highlighted in red.

The molecular effect of the missense variants was further analyzed by protein modeling using I-TASSER (Figure 3). Two pairs of compound heterozygous variants (c.8744A > G/p.N2915S and c.11689C > T/p.L3897F, c.12391_12392delinsTT/p.E4131L and c.10315C > T/p.R3439W) had hydrogen bonding changes in both biallelic variants. Another three pairs (c.3362G > A/p.S1121N and c.8680G > A/p.A2894T, c.10102G > A/p.D3368N and c.5212C > T/p.L1738F, c.1966C > G/p.L656V and c.4817C > G/p.T1606S) had hydrogen bonding changes in one of the paired variants. The other three pairs (c.5401C > T/p.P1801S and c.6878C > T/p.P2293L, c.3587C > T/p.T1196M and c.10733C > T/p.A3578V, c.6706T > C/p.F2236L and c.10760C > T/p.A3587V) showed no hydrogen bond changes, but P1801S and F2236L were predicted to decrease the protein stability measured by ΔΔG.

### Clinical Features of the Cases With *PKD1* Mutations

**FIGURE 4:**
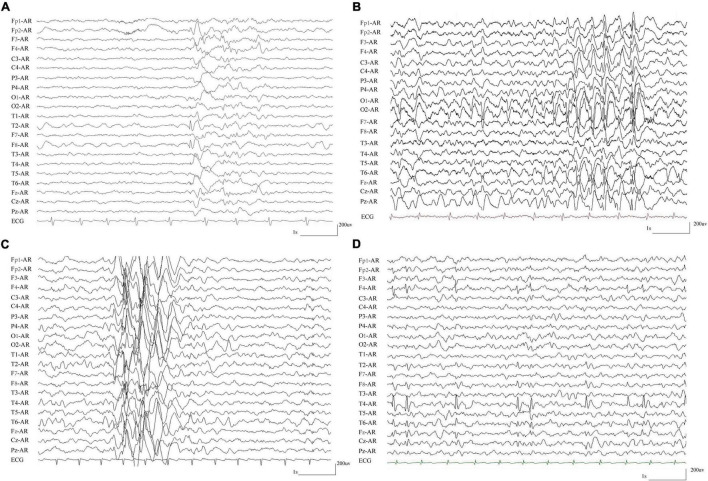
Representative EEG recordings from patients with compound heterozygous *PKD1* mutations. **(A)** Interictal EEG in case 3 showed spike-slow and slow waves in the right anterior frontal and temporal regions. **(B)** Interictal EEG in case 4 showed spike-slow and slow waves in the bilateral occipital lobes and diffused spike-slow waves. **(C)** Interictal EEG in case 5 showed irregular diffused spike-slow waves with predominance in the right areas. **(D)** Interictal EEG in case 8 showed spike-slow waves in the right frontal and temporal regions.

The detailed clinical characteristics of the eight cases with compound heterozygous *PKD1* variants were summarized in Table 1. All patients began with FS, which started ranged from 1 to 6 years old, with a median onset age of 2.7 years. Seven patients developed afebrile seizures and were diagnosed as epilepsy with antecedent FS, and one patient (case 7) was affected by only FS. Six of the patients had complex partial seizures. Five patients showed generalized tonic-clonic seizures (GTCS), whom all had one of paired variants that was located in the PKD domains. All patients suffered infrequent seizures that ranged from several episodes to monthly seizures and became seizure-free finally, including two without AEDs treatment (case 2 and 7), four after monotherapy of AEDs (case 1, 5, 6, and 8), and two with combination of two AEDs (case 3 and 4). The EEGs in the seven patients with epilepsy showed focal discharges or diffused discharges with predominance in focal regions (Table 1 and Figure 4). Brain MRI was normal in all cases. All the patients had normal development and showed no enlarged kidneys or kidney cysts.

In summary, the patients with compound heterozygous *PKD1* variants showed several common features: all began with febrile seizures; suffered infrequent seizures and became seizure-free; focal discharges in EEGs; and normal neurodevelopment.

### The Expression Profile of *PKD1*

**FIGURE 5:**
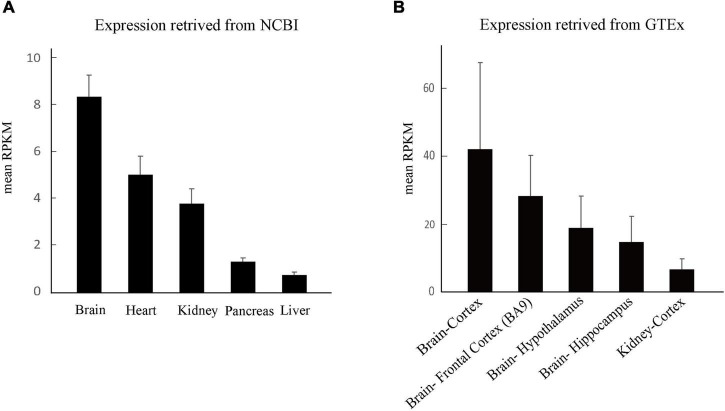
Tissue expression of *PKD1*. **(A)** The overall expression of *PKD1* in different tissues retrieved from NCBI. **(B)** Comparison of *PKD1* expression in the sub-regions of human brain and kidney retrieved from GTEx.

The expression of PC1 is ubiquitously distributed and developmentally regulated, predominantly during embryonic, infant, and adult stage. Tissue-specific expression is the basis of gene function and subsequently the clinical phenotype. We thus compared the expression of *PKD1* in the human brain and the kidney. In UniGene database, *PKD1* in the brain is expressed 1.79 times as much as that in the kidney (Figure 5A)^8^. Furthermore, data from the GTEx database showed that *PKD1* was highly and widely expressed in all sub-regions of the brain, including the cortex, hypothalamus and hippocampus. Expression of *PKD1* in these sub-regions of the brain was higher than that in the kidney, for example, the expression in the brain cortex was 6.10 times higher than that in the kidney cortex (Figure 5B)^9^.

### Genotype-Phenotype Association of *PKD1* Variants

To explore the mechanism underlying phenotypic variations, we systematically reviewed *PKD1* mutations and analyzed correlations between genotypes and phenotypes. To date, a total of 2599 mutations have been registered to be associated with ADPKD or sporadic PKD in the HGMD database and ADPKD Mutation Database. ADPKD/PKD-associated *PKD1* mutations were mainly monoallelic mutations (90.9%, 2360/2599), among which majority (72.3%, 1706/2360) were destructive mutations, including nonsense, splicing defects, frameshifting, in frame deletion/insertions, and large rearrangements. A small portion (9.1%) of the 2599 mutations were reported as biallelic mutations in ADPKD/PKD, including 114 pairs of compound heterozygotes and nine pairs of homozygotes. It was noticed that the nine pairs of *PKD1* homozygotes were associated with a severe phenotype in 14 individuals, and 21.4% of the patients with homozygotes (3/14) were premature neonate died and others (71.4%, 10/14) exhibited *in utero* onset ADPKD (Cornec-Le Gall et al., 2018; Al-Hamed et al., 2019; Garel et al., 2019; Durkie et al., 2021).

In contrast, in the present study, FS/EFS+ associated *PKD1* mutations were all compound heterozygous missense mutations (100%, 16/16).

### Evaluation of Epilepsy as a Novel Phenotype of *PKD1* Variants

We evaluated the *PKD1*-epilepsy correlation by using ClinGen Clinical-Validity Framework. The total allowable points from clinical-genetic aspect were 7.5 points and that from experimental aspect were 6 points. The results of clinical validity summary matrix were 13.5 points that was categorized as “Strong,” supporting the association between *PKD1* variants and FS/EFS+ (Table 3).

**TABLE 3:** Evaluating the clinical validity of *PKD1*-epilepsy associations based on the framework developed by the clinical genome resource.

Case-level data	Evidence type		Case information			Suggested points/case		Points given	Max score
						Default	Range		
	Variant evidence	Autosomal dominant OR X-linked disorder	Variant is *de novo*			2	0–3	0	12
			Proband with predicted or proven null variant			1.5	0–2	0	10
			Proband with other variant type with some evidence of gene impact			0.5	0–1.5	0	7
		Autosomal recessive	Two variants in trans and at least one *de novo* or a predicted/proven null variant			2	0–3	0	12
			Two variants (not predicted/proven null) with some evidence of gene impact in trans			1	0–1.5	7.5^a^	
	Segregation evidence		Evidence of segregation in one or more families	LOD score example	3	5	0–7	0	7
					2	4			
					1.5	3			
					1	0.5			

**Case-control data**	**Case-control study type**		**Case-control quality criteria**			**Suggested points/study**		**Points given**	**Max score**

	Single variant analysis		• Variant detection methodology • Power • Bias and confounding factors • Statistical significance			0–6			12
	Aggregate variant analysis					0–6		6^b^	

**Total allowable points for genetic evidence**								7.5	12

**Evidence category**			**Evidence type**			Suggested points		Points given	Max score
						Default	Range		

Function			Biochemical function			0.5	0–2	0.5^c^	2
			Protein interaction				0–2	0.5^d^	
			Expression				0–2	1^e^	
Functional alteration			Cells from affected individual			1	0–2	0	2
			Engineered cells			0.5	0–1	0	
Models and rescue			Animal model			2	0–4	3^f^	4
			Cell culture model system			1	0–2	1^g^	
			Rescue in animal model			2	0–4	0	
			Rescue in engineered equivalent			1	0–2	0	
**Total allowable points for experimental evidence**						6		6	6
**Clinical validity summary matrix**								13.5	Strong

*^a^Two pairs of compound heterozygous variants had hydrogen bonding changes and were predicted to be damaging in both biallelic variants; three pairs had hydrogen bonding changes in one variant and free energy stability changes (ΔΔG) or predicted damaging effect in the other paired variant; three pairs had free energy stability changes (ΔΔG) or predicted damaging effect in one of the paired variants (1.5 pt × 2 cases + 1.0 pt × 3 cases + 0.5 pt × 3 case = 7.5 pts).*

*^b^This is an aggregate analysis. Comparing to allele number in gnomAD-control populations and in controls of gnomAD-East Asian populations, the frequency of the variants in the present cohort is significant higher (Table 2) (Assigned 6 pts). The points are not included in total allowable points for genetic evidence.*

*^c^PC1plays multiple roles in cell proliferation, apoptosis, cell polarity, and cation transport, which is involved in neuronal excitation and synaptic plasticity (Assigned 0.5 pt).*

*^d^PC1 interacts with PC2 to form a voltage-regulated Ca^2+^ channel that regulates calcium homeostasis. As a receptor, PC1 is involved in multiple signaling pathways, such as Wnt signaling pathway, AP-1, PI3kinase/Akt, GSK3β, STAT6, and mTOR pathway, regulating cell proliferation, differentiation, and apoptosis (Assigned 0.5 pt).*

*^e^PC1 is ubiquitously distributed and highly expressed in the brain (Assigned 1 pt).*

*^f^Homozygous Pkd1 knockout mice have exhibited neural tube defects and embryonic or perinatal lethality (Assigned 3 pt).*

*^g^In human cells, mitochondrial abnormalities were identified both in the cells with homozygous PKD1 mutation and in those carrying heterozygous PKD1 mutation (Assigned 1 pt).*

## Discussion

*PKD1* encodes PC1, a large transmembrane protein that plays multiple roles in cell proliferation, apoptosis, cell polarity, and cation transport (Paul and Vanden Heuvel, 2014). Previous studies have demonstrated that *PKD1* is the major causative gene of ADPKD, of which mutations are responsible for 85% of ADPKD cases (Paul and Vanden Heuvel, 2014; Kim and Park, 2016). However, the expression of PC1 in the brain is much higher than that in the kidney (Figures 5A,B), suggesting a role in the function of the brain. In the present study, compound heterozygous *PKD1* mutations were identified in eight unrelated cases with FS and epilepsy with antecedent FS. The frequency of the *PKD1* variants in the cohort of epilepsy was significantly higher than that in control populations in gnomAD. These findings suggested that *PKD1* was potentially associated with epilepsy. The evaluation from ClinGen Clinical-Validity Framework also supports a strong association between *PKD1* mutations and epilepsy.

The transmembrane protein PC1 comprises a large extracellular N terminus, eleven membrane-spanning domains, and a short cytoplasmic C terminus. It functions as a channel subunit, cell surface receptor, and G-protein coupled receptor (Hu and Harris, 2020). PC1 may form ion channel pore by itself or contribute to channel pore *via* formation of heteromultimeric channels with PC2 (Babich et al., 2004). Generally, PC1 interacts with PC2 through the C-terminal tail to form a voltage-regulated Ca^2+^ channel, which regulates intracellular calcium influx. The simultaneous presence of both PC1 and PC2 amplify the inositol trisphosphate-induced Ca^2+^ release (Mekahli et al., 2012). Expression of PC1 can also regulate channel activity independent of the channel activity of PC2 (Babich et al., 2004). As a constitutive activator of G-proteins, PC1 activates endogenous voltage-activated Ca^2+^ channels and G protein-activated inward rectifying K^+^ channels in sympathetic neurons (Delmas et al., 2002). Deletion of PC1 caused complete loss of ionic currents (Babich et al., 2004). The calcium homeostasis is critical for neuronal stability and excitation, and the intracellular Ca^2+^ is essential for many basic cellular processes of neurons. Besides, PC1 is extensively expressed in the brain, especially in the cortex and hippocampus. These findings provide an electrophysiological and an anatomical basis for epileptogenesis.

As a receptor, PC1 is involved in multiple signaling pathways, such as Wnt signaling pathway, AP-1, PI3kinase/Akt, GSK3β, STAT6, and the mammalian target of rapamycin (mTOR) pathway (Boca et al., 2007; Boletta, 2009; Holditch et al., 2019). PC1 inhibits mTOR activity by stabilizing the tuberous sclerosis 1-tuberous sclerosis 2 (TSC1–TSC2) complex, which is known as a negative regulator of the mTOR complex (Boletta, 2009; Distefano et al., 2009). Through mediating signaling, PC1 regulates cell proliferation, differentiation, and apoptosis (Paul and Vanden Heuvel, 2014). Homozygous *Pkd1* knockout mice have exhibited neural tube defects and embryonic lethality (Lu et al., 2001; Lantinga-van Leeuwen et al., 2007), indicating that *PKD1* is important for the development of the brain. In the present study, the seven patients with EFS+ and *PKD1* mutations had complex partial seizures and/or focal discharges in EEGs, suggesting potential neurodevelopmental abnormalities. However, whether the *PKD1* variants were associated with brain malformation warrants further studies with large cohorts and advanced neuroimaging techniques.

It was noticed that five patients with GTCS had one of the paired missense variants that was located in the Ig-like PKD repeat domains of the N terminus. The PKD repeat domains play an important role in the PC1-dependent channel activity (Babich et al., 2004), possibly through regulating homophilic cell-cell/cell-matrix interactions (Ibraghimov-Beskrovnaya et al., 2000). Extracellular application of antibodies against the Ig-like PKD domains disrupted cell-cell adhesion, and reduces PC1-dependent ionic currents (Ibraghimov-Beskrovnaya et al., 2000; Babich et al., 2004). These data suggested that mutations in the PKD domains were possibly associated with GTCS, which warrants further validation by large cohorts and functional studies.

Previously reported *PKD1* mutations were mainly associated with ADPKD. From the genotype aspect, ADPKD-associated *PKD1* mutations were mainly monoallelic, and majority of the mutations were destructive (Sandford, 2009; Cornec-Le Gall et al., 2014; Lanktree et al., 2021), implying that monoallelic mutations with haploinsufficiency of *PKD1* potentially caused kidney disease. Less commonly, truncating *PKD1* mutations were identified in families ADPKD (Lanktree et al., 2021). Homozygotes of *PKD1* mutations caused severe phenotype with *in utero* onset ADPKD or premature neonate death (Cornec-Le Gall et al., 2018; Al-Hamed et al., 2019; Garel et al., 2019; Durkie et al., 2021), consistent with the embryonic lethality in homozygous *Pkd1* knockout mice (Lu et al., 2001; Lantinga-van Leeuwen et al., 2007). A quantitative correlation between genetic impairment and phenotypic severity was suggested. In the present study, the *PKD1* mutations identified in our patients with FS/EFS+ were all compound heterozygous missense mutations. All patients showed infrequent seizures and became seizure-free without AEDs treatment or after treatments of one or two AEDs. All the children showed no enlarged kidney or kidney cysts at present. Whether they will develop kidney abnormalities in later adulthood needs further follow-up. On the other hand, attention should be paid to whether the PKD patients with compound heterozygous variants had self-limited FS or mild seizures in their early life.

This study has several limitations. The direct functional effects of the mutations were not examined. The whole spectrum of phenotype of *PKD1* mutations warrants further determination with large cohorts.

## Conclusion

This study identified eight pairs of compound heterozygous missense mutations in patients with FS/EFS+. These mutations presented significantly higher frequency in case cohort than that in the control populations. Taken together the data from gene expression profile, gene functions, and *PKD1* deficiency animal model, it is suggested that *PKD1* was potentially a novel cause of epilepsy. Further analysis revealed that monoallelic mutations with haploinsufficiency of *PKD1* were associated with PKD, homozygotes with complete loss of PC1 would be embryonically lethal, whereas compound heterozygotes with superimposed effects of two missense mutations were potentially associated with epilepsy with good prognosis. The genotype-phenotype correlation helps explaining phenotypical variations.

## Data Availability Statement

The original contributions presented in the study are publicly available. This data can be found here: NCBI, OM969823, and OM969870.

## Ethics Statement

The studies involving human participants were reviewed and approved by the Ethics Committee of The Second Affiliated Hospital of Guangzhou Medical University. Written informed consent to participate in this study was provided by the participants’ legal guardian/next of kin. Written informed consent was obtained from the minor(s)’ legal guardian/next of kin for the publication of any potentially identifiable images or data included in this article.

## Author Contributions

NH, J-YW, and JW contributed to the conception of the study, interpretation of clinical data, and drafting of the manuscript. X-GL, WS, SL, D-FZ, L-DH, QP, YT, L-DG, and W-PL examined the patients and participated in drafting of the manuscript. W-PL provided critical review and substantially revised the manuscript. All authors read and approved the manuscript before sending the manuscript to the journal for publication.

## Conflict of Interest

The authors declare that the research was conducted in the absence of any commercial or financial relationships that could be construed as a potential conflict of interest.

## Publisher’s Note

All claims expressed in this article are solely those of the authors and do not necessarily represent those of their affiliated organizations, or those of the publisher, the editors and the reviewers. Any product that may be evaluated in this article, or claim that may be made by its manufacturer, is not guaranteed or endorsed by the publisher.

## Footnotes

1
https://www.omim.org/

2
http://gnomad-sg.org/

3
https://zhanglab.ccmb.med.umich.edu/I-TASSER/

4
https://folding.biofold.org/i-mutant/i-mutant2.0.html

5
http://www.hgmd.cf.ac.uk/ac/index.php

6
http://pkdb.pkdcure.org

7
http://www.uniprot.org

8
https://www.ncbi.nlm.nih.gov/gene/5310

9
https://www.gtexportal.org/home/index.html
