# Supplementary material for: Recessive PKD1 Mutations Are Associated With Febrile Seizures and Epilepsy With Antecedent Febrile Seizures and the Genotype-Phenotype Correlation
Source: Front Mol Neurosci. 2022 May 10;15:861159. doi: 10.3389/fnmol.2022.861159 (PMC9128595; doi:10.3389/fnmol.2022.861159)
Supplement: Supplementary file 1 [file Table_1.DOCX]

**Supplementary Data**

**Table S1. Bioinformatics Analysis of the** ***PKD1* Mutations**

| **Case** | **cDNA change (NM_014689.2)** | **Protein change** | **Inheritance** | **MAF** | **MAF-EAS** | **SIFT** | **PP2_Var** | **Mutation-Taster** | **CADD** | **Fathmm-MKL** | **FitCons** | **GERP++** | **PhastCons** |
| --- | --- | --- | --- | --- | --- | --- | --- | --- | --- | --- | --- | --- | --- |
| Case 1 | c.3362G>A  c.8680G>A | p.S1121N  p.A2894T | Paternal  Maternal | 0  0.00008908 | 0  0 | T (0.251)  T (0.231) | B (0.434)  B (0.148) | P (1.0)  P (1.0) | T (0.060)  T (10.06) | T (-0.3)  T (-0.62) | D (0.707)  D (0.707) | C (3.53)  NC (-4.11) | NC (0.021)  NC (0.0) |
| Case 2 | c.6878C>T  c.5401C>T | p.P2293L  p.P1801S | Paternal  Maternal | 0.0001525  0.00003771 | 0.001044  0.0003429 | T (0.655)  T (0.093) | B (0.185)  PD (0.992) | P (0.991)  P (0.740) | T (8.095)  D (23.5) | T (-0.38)  T (-0.38) | D (0.660)  D (0.672) | NC (1.8)  C (4.71) | C (1.000)  C (1.000) |
| Case 3 | c.8744A>G  c.11689C>T | p.N2915S  p.L3897F | Paternal  Maternal | 0.0001540  0.0009823 | 0.001041  0 | D (0.021)  D (0.048) | B (0.238)  PD (0.964) | P (0.999)  P (0.701) | T (18.84)  D (24.1) | T (-0.7)  T (-0.54) | D (0.707)  T (0.685) | C (4.89)  C (2.95) | C (1.000)  C (1.000) |
| Case 4 | c.12391_12392delinsTT  c.10315C>T | p.E4131L  p.R3439W | Paternal  Maternal | 0  0.0001715 | 0  0.001841 | -  D (0.001) | -  PD (0.93) | -  P (1.000) | -  D (24.5) | -  T (1.06) | -  T (0.672) | -  NC (0.003) | -  NC (0.000) |
| Case 5 | c.3587C>T  c.10733C>T | p.T1196M  p.A3578V | Paternal  Maternal | 0.00003801  0.00002481 | 0.0005054  0.0001425 | D (0.003)  T (0.657) | PD (0.988)  B (0.043) | P (1.000)  P (1.000) | D (20.3)  T (0.240) | D (0.840)  T (0.058) | T (0.646)  T (0.672) | C (2.33)  C (3.01) | C (1.000)  NC (0.000) |
| Case 6 | c.10102G>A  c.5212C>T | p.D3368N  p.L1738F | Paternal  Maternal | 0.0002220  0.00004743 | 0.002514  0.0003814 | T (0.108)  T (0.095) | B (0.275)  B (0.072) | D (1.000)  P (1.0) | D (24.4)  T (0.003) | D (0.970)  D (0.531) | D (0.707)  T (0.672) | C (3.34)  NC (-2.74) | C (1.000)  NC (0.981) |
| Case 7 | c.6706T>C  c.10760C>T | p.F2236L  p.A3587V | Paternal  Maternal | 0.00001841  0.00003399 | 0.0002306  0 | T (0.22)  D (0.019) | PD (0.997)  B (0.036) | D (0.987)  P (0.996) | D (24.1)  D (20.2) | D (0.987)  T (0.173) | T (0.635)  T (0.635) | C (4.27)  NC (0.079) | C (1.000)  C (1.000) |
| Case 8 | c.1966C>G  c.4817C>G | p.L656V  p.T1606S | Paternal  Maternal | 0  0.00001828 | 0  0.0002211 | T (0.346)  D (0.032) | B (0.23)  PD (0.998) | P (1.0)  D (1.000) | D (24.1)  D (25.0) | T (0.632)  D (0.985) | D (0.707)  D (0.707) | C (2.18)  C (5.12) | NC (0.010)  C (1.000) |

B, benign; C, conserved; CADD, combined annotation dependent depletion; D, damaging; FitCons, the fitness consequences of functional annotation; MAF, minor allele frequency from gnomAD (controls); MAF-EAS, minor allele frequency from gnomAD (controls)-East Asian population; NA, not available; NC, non-conserved; P, polymorphism; PD, probably_damaging; PP2_Var, polyphen2_HVAR; T, tolerable.
